# Predicting habitat suitability for an endangered medicinal plant, *Saussurea medusa*: insights from ensemble species distribution models

**DOI:** 10.3389/fpls.2025.1590206

**Published:** 2025-06-24

**Authors:** Xiang Guo, Wei Bai, Yihua Wang, Senliang Hao, Liping Zhao, Xin Li, Zongliang Guo, Xiaoyan Li

**Affiliations:** ^1^ Shanxi Bethune Hospital, Shanxi Academy of Medical Sciences, Third Hospital of Shanxi Medical University, Tongji Shanxi Hospital, Taiyuan, China; ^2^ Department of Radiation Oncology, Shanxi Province Cancer Hospital, Taiyuan, China; ^3^ Department of Oncology, Taiyuan Central Hospital, Taiyuan, China; ^4^ Guizhou medical university pharmacy college Pharmacy (Chinese-foreign cooperation in running schools), Guiyang, China; ^5^ Department of Pathology, Shanxi Provincial People’s Hospital, Taiyuan, China; ^6^ Department of Geriatrics, Shanxi Provincial People’s Hospital, Taiyuan, China; ^7^ Department of Gastrointestinal Surgery, Shanxi Province Cancer Hospital, Taiyuan, China; ^8^ Department of Clinical Laboratory, Heping Branch, Shanxi Provincial People’s Hospital, Taiyuan, China; ^9^ Department of Blood Transfusion, Shanxi Provincial People’s Hospital, Taiyuan, China

**Keywords:** Saussurea medusa, species distribution modeling, habitat distribution, climate change, medicinal plants

## Abstract

**Introduction:**

Global climate change has profound impacts on alpine ecosystems, and with climate warming, alpine plants often face a substantial risk of habitat loss. *Saussurea medusa* Maxim., known for its significant medicinal value, is a typical alpine plant predominantly found in the high-altitude regions of Southwest China. However, the impacts of climate change on the habitat suitability of *S. medusa* have not been fully understood.

**Methods:**

We simulated ensemble species distribution models to assess the spatiotemporal habitat distribution pattern of *S. medusa* under different climate scenarios (ssp126 and ssp585) for the periods 2040s, 2060s, and 2070s.

**Results and Discussion:**

The results show that the suitable habitats, under near current condition, are mainly distributed in the Qinghai-Xizang Plateau of China, covering the border regions of four provinces: Xizang, Qinghai, Sichuan, and Yunnan, with a total area of 14.06×10^4^ km^2^. Under future climate change scenarios, the area of suitable habitats, particularly the highly suitable habitats, is projected to contract significantly by 80.65%, accompanied by shifts in distribution centroids towards the southwest and higher altitudes in Xizang. These results indicate that the risk of *S. medusa* survival due to the loss of suitable habitats would persist in the future. Among the environmental factors analyzed, elevation and three bioclimatic predictors, BIO18 (precipitation of the warmest quarter), BIO12 (annual precipitation) and BIO4 (temperature seasonality), exhibit significant impacts on the potential distribution of suitable habitats for *S. medusa*. Our study provides an improved understanding of the potential habitat distribution dynamics of the endangered *S. medusa*, thereby offering a crucial reference for its conservation and sustainable management.

## 1 Introduction

Understanding the geographic distribution patterns of a species is imperative for comprehending its biogeography, evolution, and conservation concerns. Species distribution patterns can be influenced by various factors, such as climate, habitat availability, and interspecies interactions. Among these factors, climate-associated temperature and precipitation have been identified as significant factors (Song et al., 2023; Thomas et al., 2004; Trisos et al., 2020), potentially because of their close correlation with energy and water availability, which are crucial for species survival (Barbet-Massin and Jetz, 2014; Bell et al., 2014). With climate change, particularly climate warming, it is predictable that the distribution range of species is spatially dynamic and often shifts towards higher elevations and latitudes to track suitable habitats (Parmesan, 2007; Lenoir and Svenning, 2015; Thuiller et al., 2011).

To understand species distribution spatiotemporally, one of the most popular methods is the use of species distribution models (SDMs). These models link the ecological requirements of a species, based on its known occurrence points, to layers of environmental predictor data. Consequently, the potential distribution of the species across various habitats is projected based on similar environmental conditions (Elith and Leathwick, 2009; Neupane et al., 2024; Zhu et al., 2013). There are a series of modeling algorithms, such as generalized linear model (GLM, Nelder and Wedderburn, 1972), random forest (RF, Breiman, 2001), and the maximum entropy model (MaxEnt, Phillips et al., 2006). Nevertheless, an ensemble modeling approach, which combines a set of diverse SDMs to estimate habitat suitability through consensus, is increasingly recommended and widely applied (Duffy et al., 2017; Gan et al., 2025; Li et al., 2024a, 2024b; Song et al., 2023; Zhang et al., 2017). This is because the ensemble method offers a more robust approach than relying on a single SDM algorithm and mitigates the effects of individual model biases (Araújo and New, 2007; Grenouillet et al., 2011; Thuiller et al., 2009).

*Saussurea medusa* Maxim., a member of the Asteraceae family, is an alpine plant predominantly found in the high-altitude regions of Southwest China, including Qinghai, Gansu, Yunnan, Sichuan, and Xizang, at elevations between 3000 and 5600 meters (Liu, 1996). *S. medusa* has been widely utilized as a traditional Tibetan medicine for thousands of years, and has significant medicinal value because of its extensive pharmacological activities including anti-inflammation, anti-tumor, anti-aging, anti-hypoxia, and others (Bo et al., 2013; Fan et al., 2015; Guo et al., 2024; Qiu et al., 2010; Takasaki et al., 2000). In recent years, the population density of *S. medusa* has declined due to habitat loss caused by climate warming and over-harvesting by humans, and it has been listed as a second-class endangered plant in China. Consequently, it is crucial for conservation efforts to evaluate the distribution patterns of *S. medusa* and its response to climate change using the SDM methods. Peng et al. (2019) employed MaxEnt algorithm to predict the distribution range of *S. medusa* habitats, and found that the potential suitable areas would be reduced under future climate change scenarios. However, this study was conducted with a single model algorithm and limited distribution points, and a comprehensive investigation into the distribution dynamics of *S. medusa*, based on an ensemble model, is still needed.

In the present study, we simulated ensemble species distribution models to assess the spatiotemporal distribution pattern of *S. medusa* under climate change scenarios, as well as to identify the underlying driving factors. Furthermore, we discussed the conservation implications for *S. medusa* by comparing our predictive results with the existing distribution of National Nature Reserves in China. Our objectives were to enhance our understanding of the spatiotemporal distribution patterns of *S. medusa* under current and future environmental conditions, assess its endangered status, and thereby provide a reference for its conservation and sustainable management.

## 2 Material and methods

### 2.1 Occurrence records

**Figure 1:**
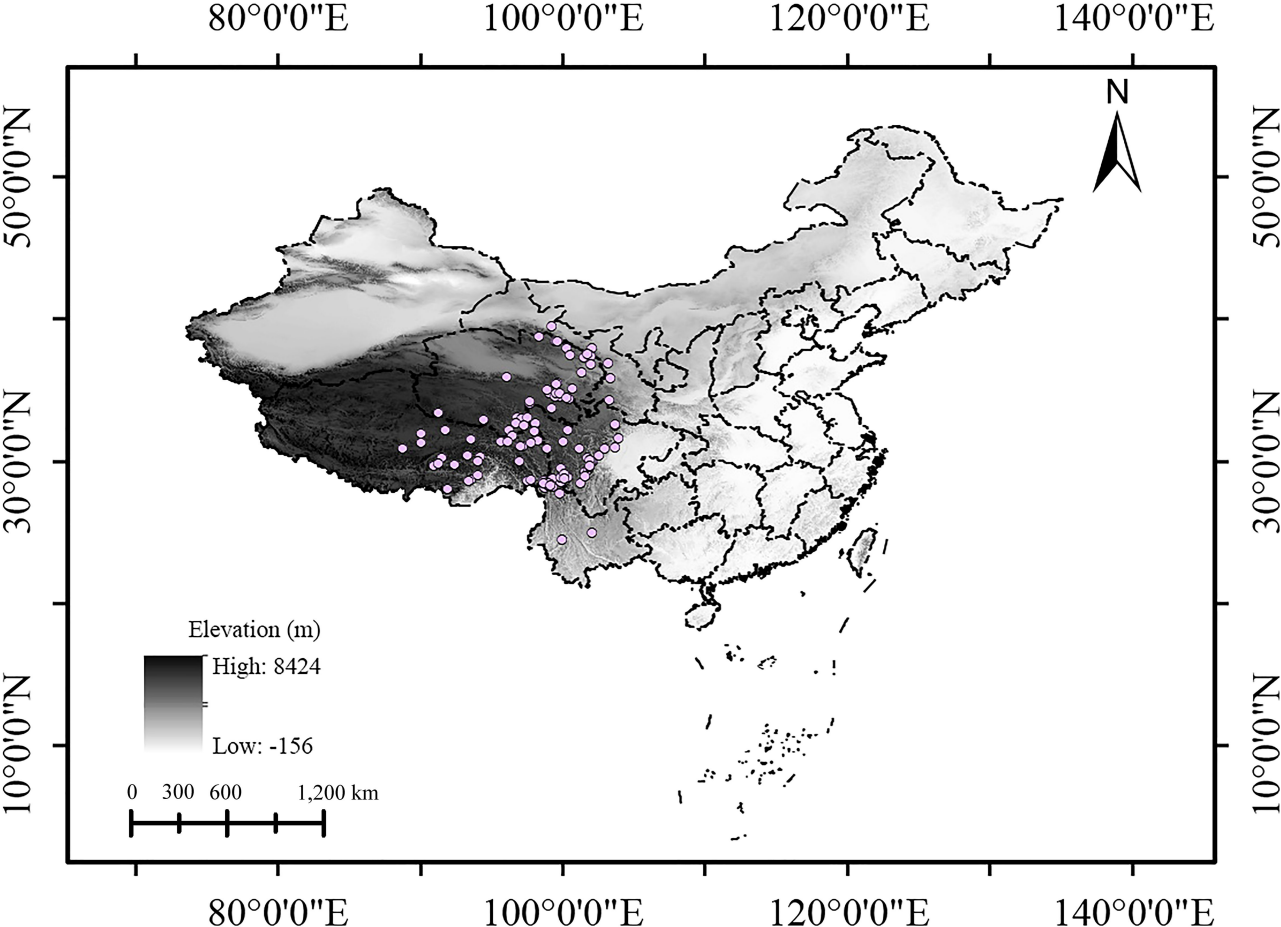
Occurrence data of *Saussurea medusa* used in the models.

Initially, a total of 209 occurrence records (**Supplementary Figure S1**, **Supplementary Table S1**), almost covering the whole known distribution range of *S. medusa*, were gathered from four sources: the global biodiversity information facility database (GBIF, https://www.gbif.org/species/5404320), iPlant (https://www.plantplus.cn/info/Saussurea%20medusa), Tropicos (https://www.tropicos.org/name/50056617), and the published literature searched in China National Knowledge Infrastructure (CNKI, https://www.cnki.net). For distribution information in the literature that was recorded with only detailed localities, the website https://api.map.baidu.com/lbsapi/getpoint/ was used to convert this information into coordinate form. Given that sampling bias could lead to model overfitting (Phillips et al., 2009; Zhu et al., 2014), the R package “spThin” (Aiello-Lammens et al., 2015) was employed to thin the occurrence points, ensuring that only one occurrence record appears in each raster cell (about 4.5 km^2^). Furthermore, where occurrence points remained densely clustered, additional manual adjustments were made. Ultimately, 131 occurrence records were implemented in the models (**Figure 1**; **Supplementary Table S2**).

### 2.2 Environmental variables

The 19 bioclimatic and elevation layers were downloaded from WorldClim 2.1 (https://www.worldclim.org/) (Fick and Hijmans, 2017). The bioclimatic data for near current conditions represented the period from 1970 to 2000. For future projections, the bioclimatic data we utilized represented three periods: 2021–2040, 2041–2060, and 2061–2080, under both low (ssp126) and high (ssp585) greenhouse gas emission scenarios from the Coupled Model Intercomparison Project 6 (CMIP6) version. For each period, the Beijing Climate Center Climate System Model (BCC-CSM2-MR) developed at the National Climate Center (Wu et al., 2019), was selected due to its extensive use in SDM studies focused on China (Gan et al., 2025; Li et al., 2024a; Zhang et al., 2021). For bioclimatic variables, the R package “corrplot” (Wei and Simko, 2021) was used to perform a Pearson’s correlation analysis, and for the two variables with a correlation coefficient |r| > 0.8, the one with a lower contribution in the initial ensemble model was removed to minimize multicollinearity among variables (Dormann et al., 2013; Zhang et al., 2022). The environmental layers selected were used in the subsequent modeling at a resolution of 2.5 arc-minutes.

### 2.3 Species distribution modeling

An ensemble model was developed using the R package “sdm” (Naimi and Araujo, 2016). Initially, an ensemble model was constructed with twelve commonly used models from the “sdm” package, namely BIOCLIM (Busby, 1991), classification and regression trees (CART, Loh, 2011), Domain (Reinhartz-Berger, 2010), flexible discriminant analysis (FDA, Hastie et al., 1994), generalized additive model (GAM, Hastie and Tibshirani, 1987), generalized linear model (GLM, Nelder and Wedderburn, 1972), Glmnet (Engebretsen and Bohlin, 2019), maximum entropy (MaxEnt, Phillips et al., 2006), Maxlike (Royle et al., 2012), multivariate adaptive regression spline (MARS, Friedman, 1991), support vector machine (SVM, Hearst et al., 1998), and random forests (RF, Breiman, 2001). In this analysis, the following parameters were implemented: the “gRandom” method of the ‘sdmData’ function to randomly generate 1000 pseudoabsence points (Barbet-Massin et al., 2012), with 75% of the distribution data used as training data and the remaining 25% as test data, employing a ten-fold cross validation approach. The maximum iterations were set to 5000 (Li et al., 2024a; Peng et al., 2019; Ran et al., 2024; Zhang et al., 2022). Consequently, the 12 single models were initially developed with the values of the area under a receiver operating characteristic (ROC) curve (AUC) (Lobo et al., 2008) and the true skill statistic (TSS) (Allouche et al., 2006). Based on the AUC and TSS values, the five top-performing single models were selected to jointly create the final ensemble models.

In the final modeling phase, the “ensemble” function was utilized to combine the output results of the selected individual models using a weighted average approach. The “roc” and “rcurve” functions were employed to generate the ROC curves for single models and response curves for each variable, respectively. To assess the impact of non-climate variables on projections, two variable combinations were independently used to develop ensemble models under current conditions: one with only bioclimatic variables (BIOs) and another with bioclimate plus elevation. For future projections, only BIOs were used, and the “ensemble” function with a weighted average approach was applied as well.

### 2.4 Model evaluation and analyses

In the ensemble model, average AUC and TSS values were generated to evaluate model performance. According to Peterson et al. (2008) and Hand and Anagnostopoulos (2013), an AUC value of 0.7–0.8 is considered acceptable, 0.8–0.9 is deemed great, and a value greater than 0.9 is remarkable. Conversely, a value less than 0.5 indicates that the model’s performance is no better than random. Additionally, the TSS value was considered because the AUC criterion can be misleading due to its equal weighting of sensitivity and specificity (Zhu et al., 2017). The TSS value ranges from −1 to +1, where +1 signifies perfect prediction, and values of zero or less indicate that the model’s performance is no better than random (Allouche et al., 2006; Pearce and Ferrier, 2000).

From the modeling, the habitat suitability was originally generated as continuous values. To characterize and compare the distribution patterns of different projections, the habitat suitability was defined into four classes using DIVA-GIS (Hijmans et al., 2001): “highly suitable” (0.6–1), “moderately suitable” (0.4–0.6), “minimally suitable” (0.2–0.4), and “unsuitable” (< 0.2) (Li et al., 2024a; Liu et al., 2022; Zhang et al., 2018). The R package “ggplot2” (Wickham, 2016) was used to visualize the response curves depicting the probability of *S. medusa* presence as environmental variable changes. OriginPro version 2021 (OriginLab Corporation, Northampton, MA, USA) was employed to visualize the percent contribution of each variable and the values of AUC and TSS generated in the models. Furthermore, the distribution data of National Nature Reserves from National Specimen Information Infrastructure (http://bhq.papc.cn/specimen.html) were mapped onto the prediction results to reflect the overlapping state, offering insights for conservation efforts.

## 3 Results

### 3.1 Variable selection, model selection and evaluation

**Figure 2:**
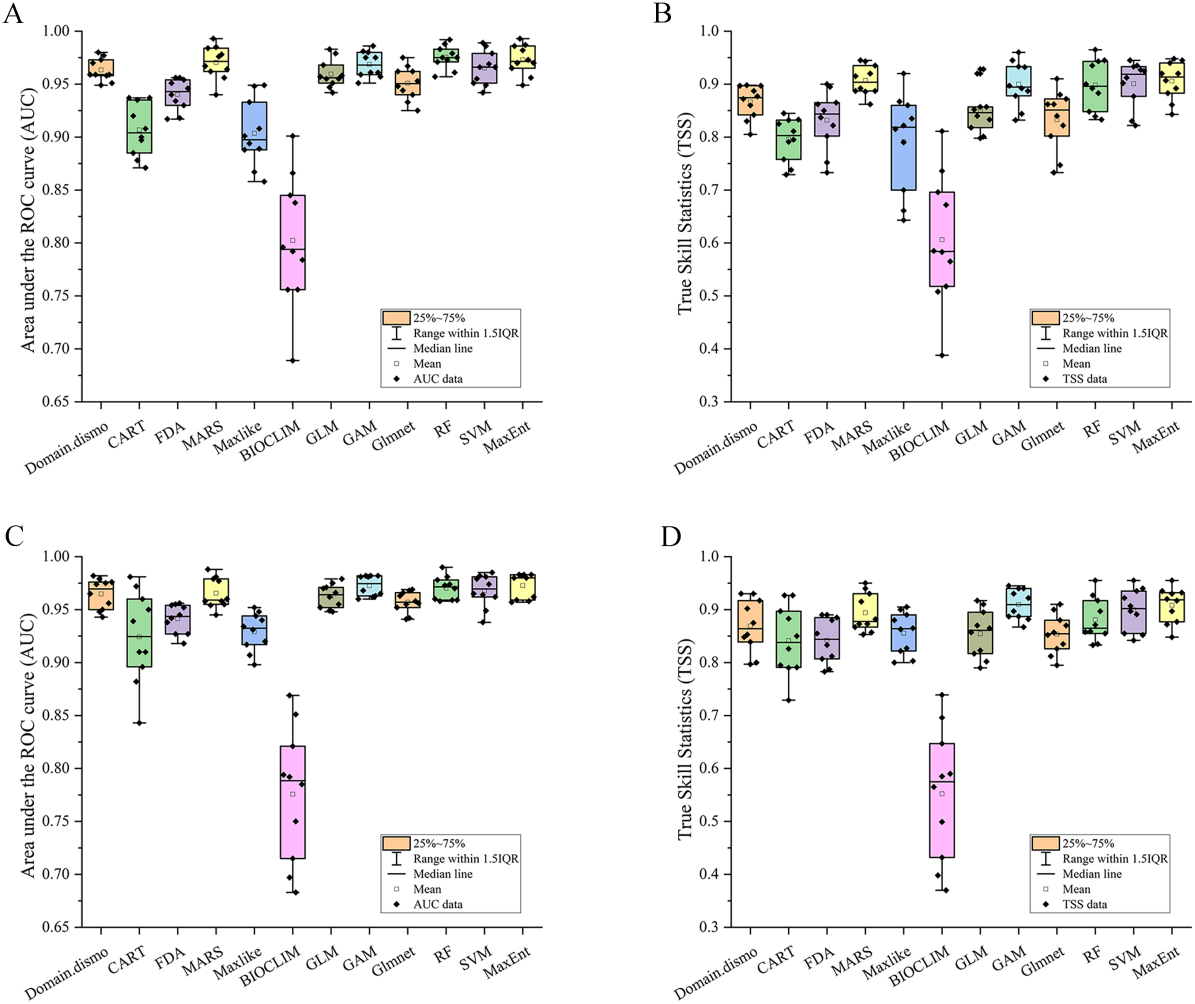
The area under the receiver operating characteristic curve (AUC) and true skill statistics (TSS) values of the twelve models. **(A)** AUC values under BIOs; **(B)** TSS values under BIOs; **(C)** AUC values under BIOs + elevation; **(D)** TSS values under BIOs + elevation.

**Figure 3:**
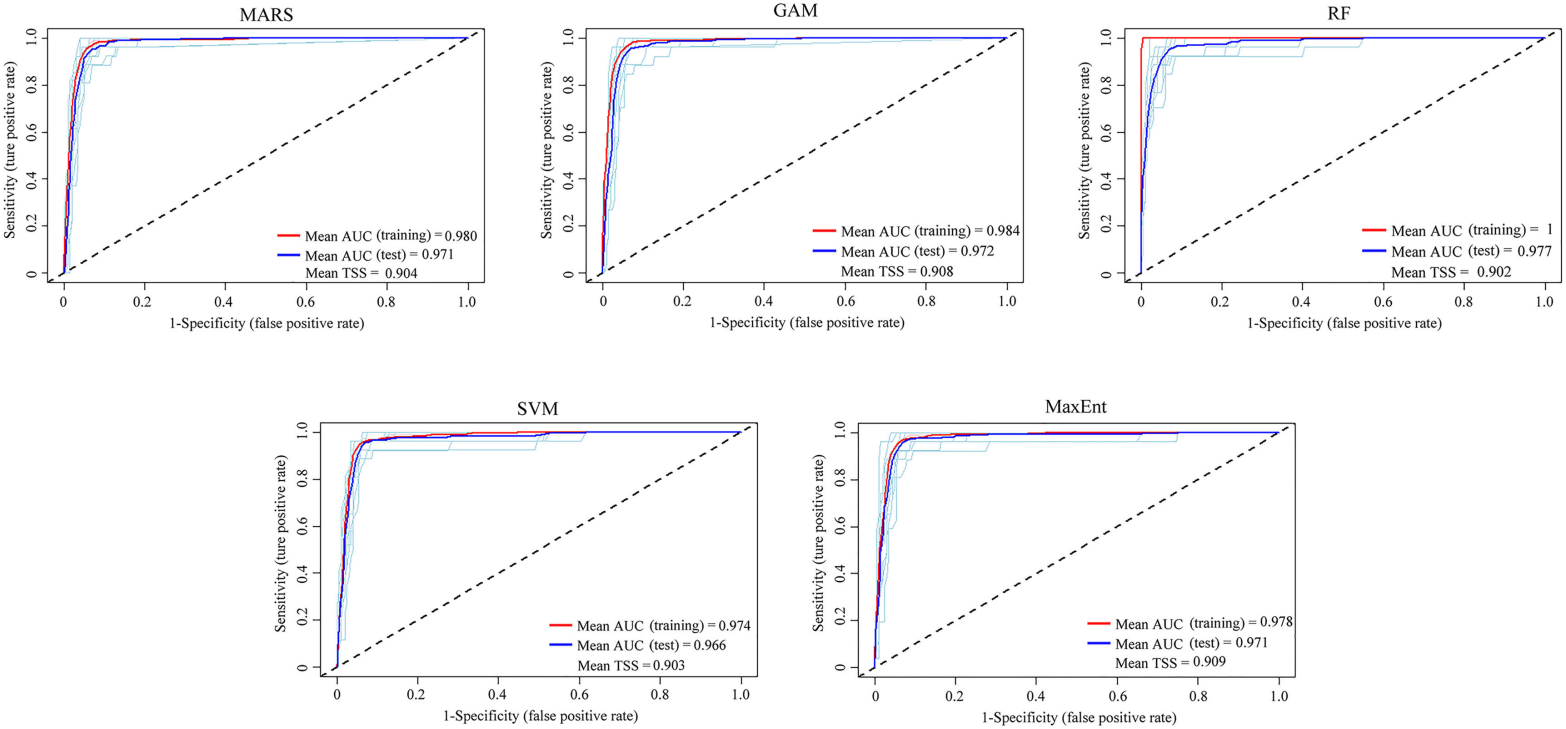
The area under the receiver operating characteristic curve (AUC) and true skill statistics (TSS) values for five single models of the ensemble models under BIOs.

Following a Pearson’s correlation analysis (**Supplementary Figure S2**) and initial model evaluation (**Figure 2**), eight of the 19 bioclimate variables (BIO2, BIO4–BIO5, BIO12, BIO15, and BIO17–BIO19) were retained, and plus one elevation, totally ten predictors were implemented in the ensemble models (**Table 1**). According to the values of AUC and TSS, the five single models (MARS, GAM, RF, SVM, and Maxent) were selected to develop ensemble models for both variable combinations of BIOs and BIOs + elevation (**Figure 2**). In the ensemble models, the average AUC and TSS values were 0.97 and 0.91 respectively, for the BIOs (**Figure 3**), and 0.97 and 0.90 for the BIOs + elevation (**Supplementary Figure S3**). This indicated that the model performances for both ensemble models were excellent and the predicted habitat suitability was reliable.

**Table 1:** The environmental variables considered in this study.

**Variable type**	**Variable**	**Description**
Bioclimatic Variables	BIO1	Annual mean temperature (°C)
	**BIO2**	Mean diurnal range (mean of monthly (max temp-min temp)) (°C)
	BIO3	Isothermality (bio2/bio7) (× 100)
	**BIO4**	Temperature seasonality (standard deviation ×100)
	**BIO5**	Max temperature of warmest month (°C)
	BIO6	Min temperature of coldest month (°C)
	BIO7	Annual temperature range (bio5–bio6) (°C)
	BIO8	Mean temperature of wettest quarter (°C)
	BIO9	Mean temperature of driest quarter (°C)
	BIO10	Mean temperature of warmest quarter (°C)
	BIO11	Mean temperature of coldest quarter (°C)
	**BIO12**	Annual precipitation (mm)
	BIO13	Precipitation of wettest month (mm)
	BIO14	Precipitation of driest month (mm)
	**BIO15**	Precipitation seasonality (coefficient of variation)
	BIO16	Precipitation of wettest quarter (mm)
	**BIO17**	Precipitation of driest quarter (mm)
	**BIO18**	Precipitation of warmest quarter (mm)
	**BIO19**	Precipitation of coldest quarter (mm)
Elevation	**elevation**	Ground height above sea level (m)

The variables in bold were finally used in the modeling.

### 3.2 Variables importance in the modeling

**Figure 4:**
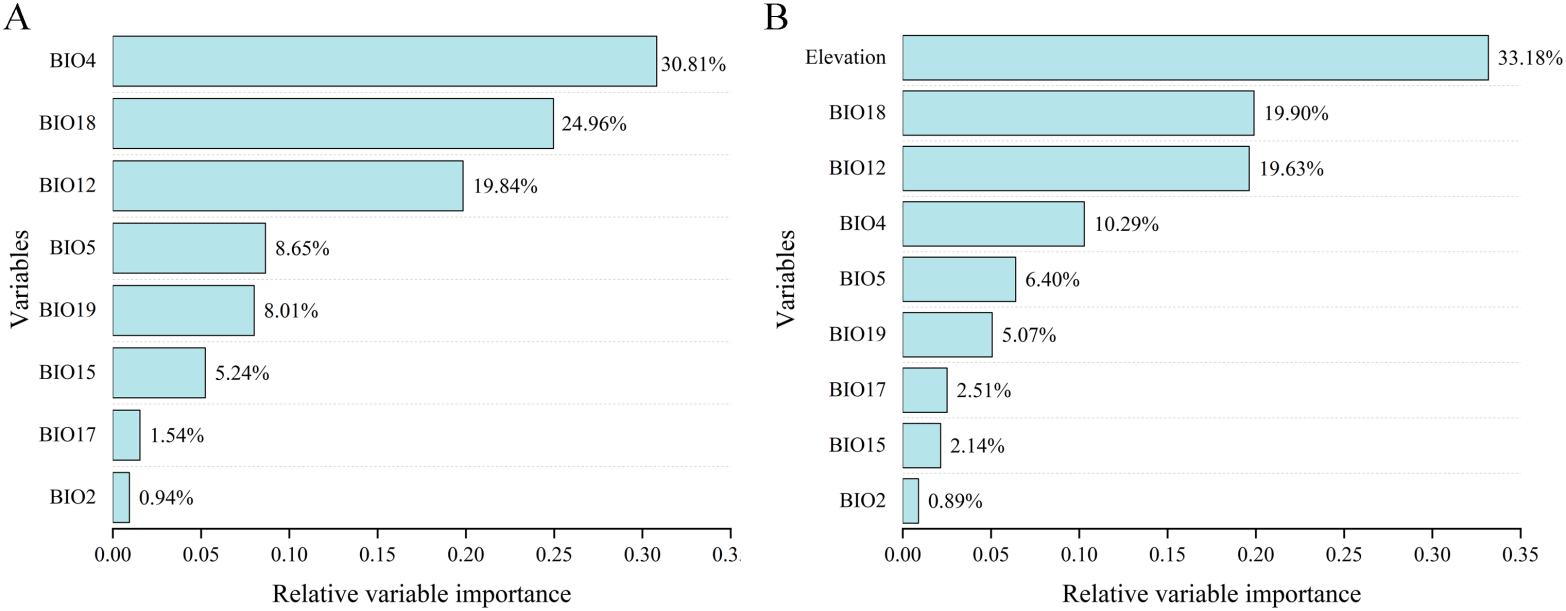
Percent contribution of environmental variables used in the models under different two variable combinations. **(A)** BIOs; **(B)** BIOs + elevation.

The percentage contribution of each variable in two ensemble models under two variable combinations is shown in **Figure 4**. Under the BIOs scenario (**Figure 4A**), BIO4 was the most significant contributor to the projection, with a contribution of 30.81%, followed by BIO18 at 24.96%, BIO12 at 19.84%, BIO5 at 8.65%, and BIO2 exhibited the lowest contribution at 0.94%. None of the nine factors used were revealed to be predominant predictors (>50%) in terms of contributing to the model. In the model under BIOs + elevation (**Figure 4B**), elevation exhibited the highest contribution at 33.18%, followed by BIO18 at 19.90% and BIO12 at 19.63%, while BIO2 had the lowest contribution at 0.89%.

### 3.3 Response curves of the variables on presence probability of *S. medusa* habitat

**Figure 5:**
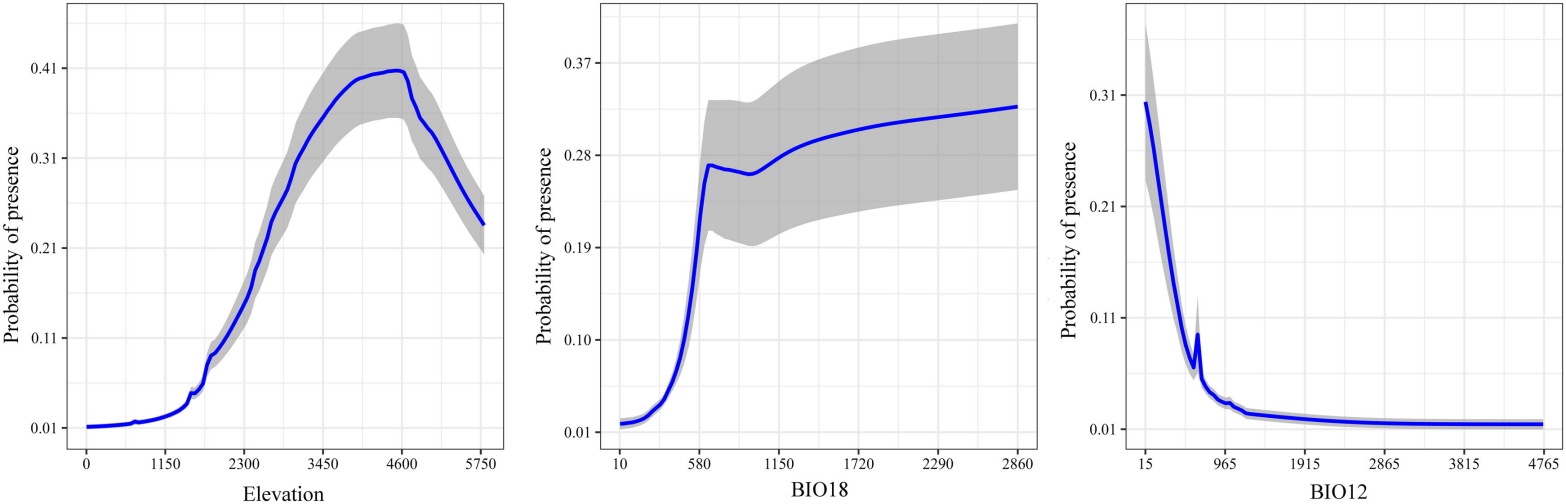
Response curves of the top three environmental variables in the modeling under BIOs + elevation.

To understand the probability of *S. medusa* presence as variable change, the response curves of the top three variables contributing to the ensemble model under BIOs + elevation are illustrated in **Figure 5**. From sea level, the probability of *S. medusa* presence increased and reached its highest value of 0.41 at an elevation of 4600 meters above sea level, after which it sharply declined with further increases in elevation. For BIO18 (precipitation of the warmest quarter), the probability sharply increased from 10 mm to 0.27 at 674 mm of precipitation, and then tended to increase very slowly. At 16 mm of BIO12 (annual precipitation), the probability reached its highest value of 0.30, initially declined sharply, and then generally remained at 0.01 as the annual precipitation increased.

### 3.4 The current potential distribution of *S. medusa* habitats

**Figure 6:**
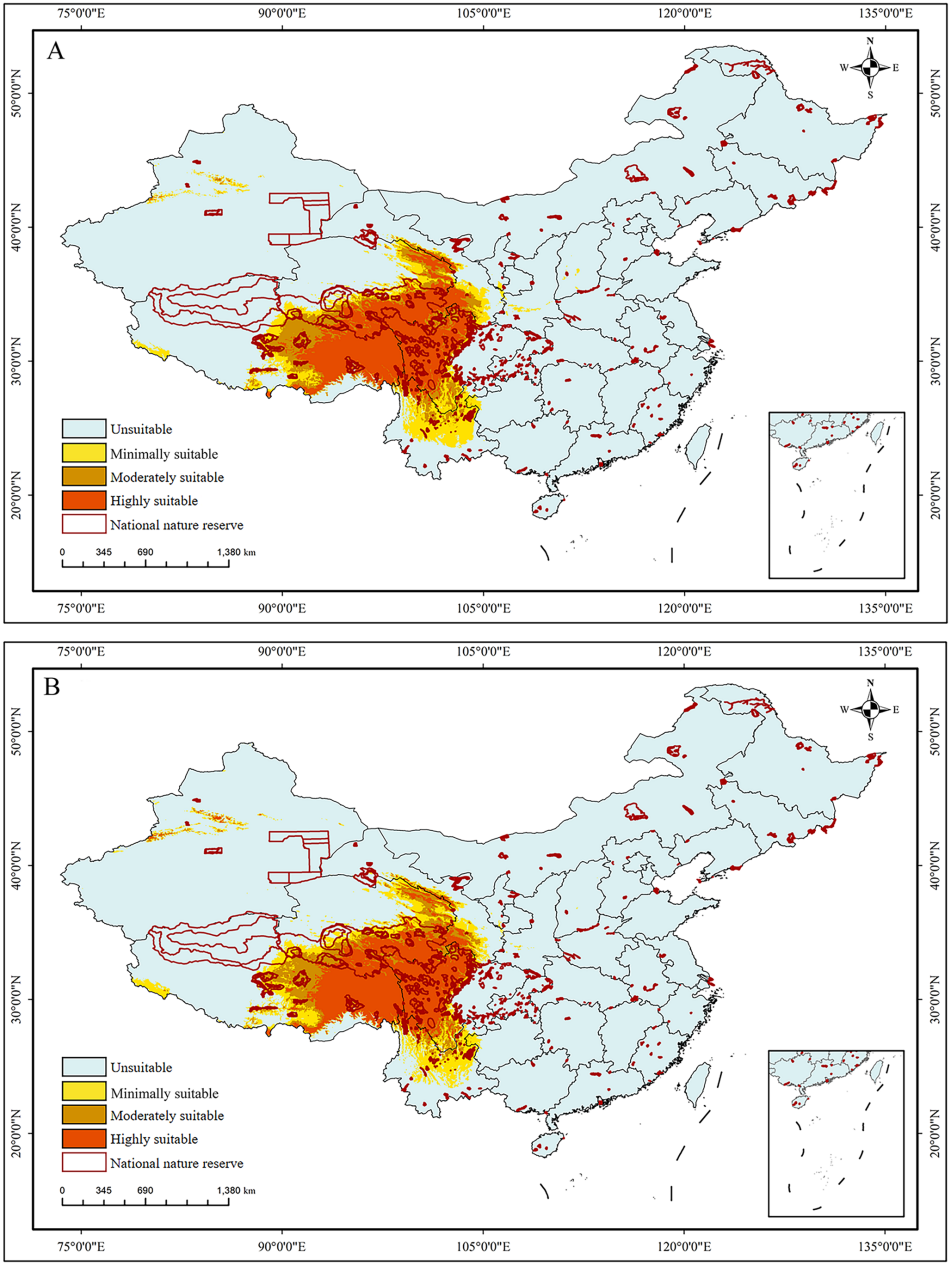
The predicted habitat suitability of *Saussurea medusa* under current environmental conditions. **(A)** BIOs; **(B)** BIOs + elevation. 0–0.2: unsuitable, 0.2–0.4: minimally suitable, 0.4–0.6: moderately suitable, 0.6–1: highly suitable.

Two projections for the current potential distributions of *S. medusa* were conducted based on different combinations of variables. Suitable habitats (**Figure 6A**), inferred under only bioclimatic variables, were widely distributed in Southwest China, mainly including East Xizang, South Qinghai, West Sichuan, and North Yunnan. The suitable habitats had a total area of 14.06×10^4^ km^2^, with the minimally, moderately, and highly suitable habitats being 4.09×10^4^ km^2^, 3.20×10^4^ km^2^, and 6.77×10^4^ km^2^, respectively (**Table 2**). When the elevation layer was added, the distributions of the suitable habitats (**Figure 6B**) predicted were generally identical to those predicted with bioclimatic variables for all three levels of suitability.

**Table 2:** The change of suitable areas of Saussurea medusa under current conditions and future climate change scenarios.

Decades/climate scenarios		Area (×104 km2)			
		All suitable habitats	Minimally suitable habitats	Moderately suitable habitats	Highly suitable habitats
1970–2000		14.06	4.09	3.20	6.77
ssp126	2030s	14.12	3.30	7.78	3.04
ssp126	2050s	13.56	3.74	7.62	2.20
ssp126	2070s	13.24	3.42	7.74	2.08
ssp585	2030s	14.08	3.30	7.99	2.79
ssp585	2050s	13.35	5.17	6.79	1.39
ssp585	2070s	13.05	5.81	5.92	1.31

### 3.5 The future potential distribution of *S. medusa* habitats

**Figure 7:**
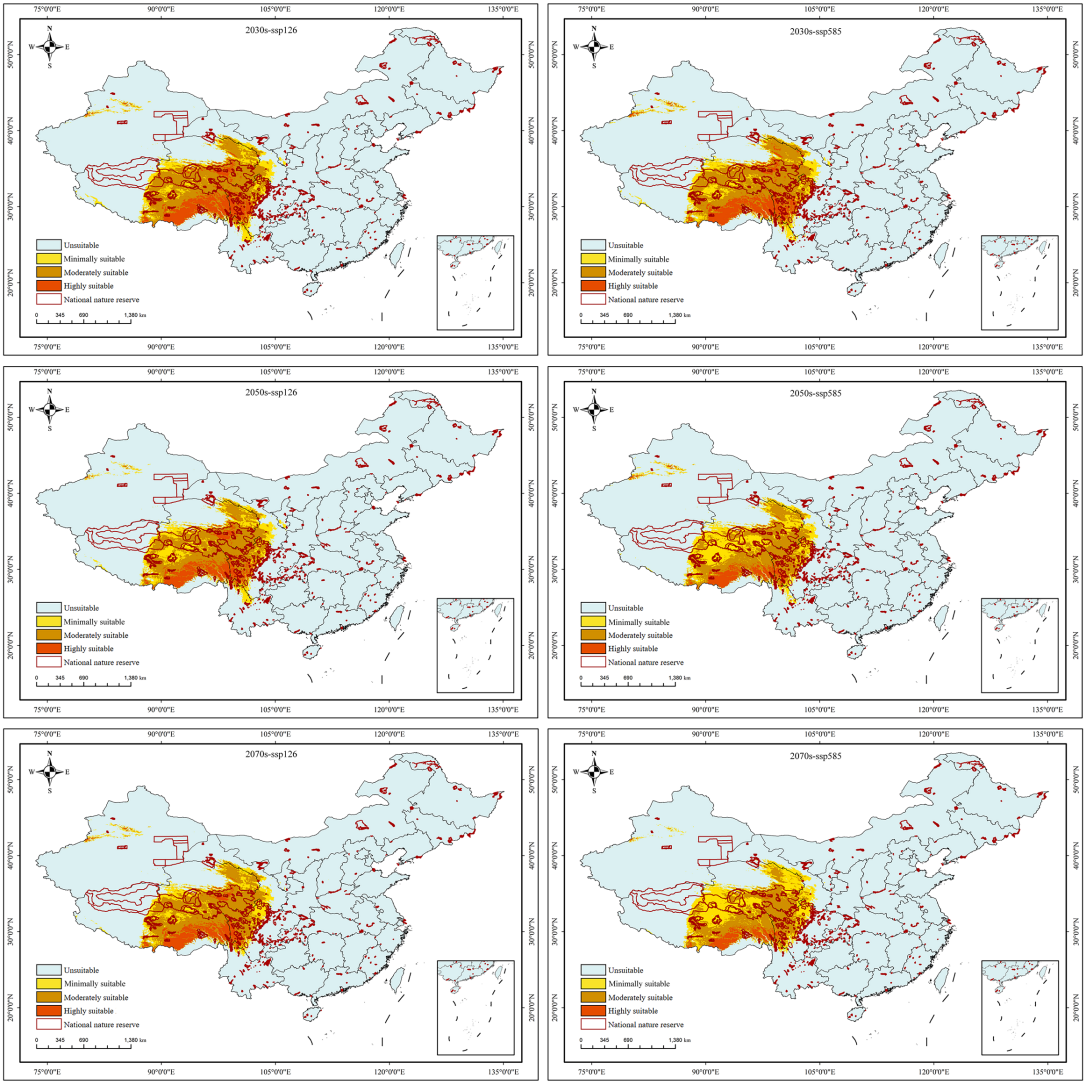
The predicted habitat suitability of *Saussurea medusa* under future climate change scenarios. 0–0.2: unsuitable, 0.2–0.4: low suitable, 0.4–0.6: moderately suitable, 0.6–1: highly suitable.

Under the BIOS, six future prediction maps (**Figure 7**) were processed using variables representing three periods and two greenhouse gas emission scenarios. In future projections, the distribution range of suitable habitats exhibited an overall movement towards the west, primarily in East Xizang, Southeast Qinghai, and West Sichuan, resulting in almost no suitable habitats in North Yunnan. Particularly, when compared to the current near-future conditions, the distribution of highly suitable habitats would undergone a significant change, only found in Southeast Xizang. Overall, the area of suitable habitats for *S. medusa* would slightly decrease under future climate change scenarios, with an average of 13.57×10^4^ km^2^, compared to the 14.06×10^4^ km^2^ under near current condition. However, the proportions of minimally, moderately, and highly suitable habitats would differ significantly (**Table 2**). In comparison to the 6.77×10^4^ km^2^ under near current condition, the highly suitable habitats would have a significant contraction. At ssp585 of 2070s, there was only 1.31×10^4^ km² distributed in Southeast Xizang. Conversely, the area of moderately suitable habitats were expanded in all future climate change scenarios (7.31×10^4^ km^2^ on average). For the minimally suitable habitats, the areas were expanded at ssp585 of 2070s and 2050s, and contracted under other future climate change scenarios.

### 3.6 Centroid shifts of *Saussurea medusa* in future climatic scenarios

**Figure 8:**
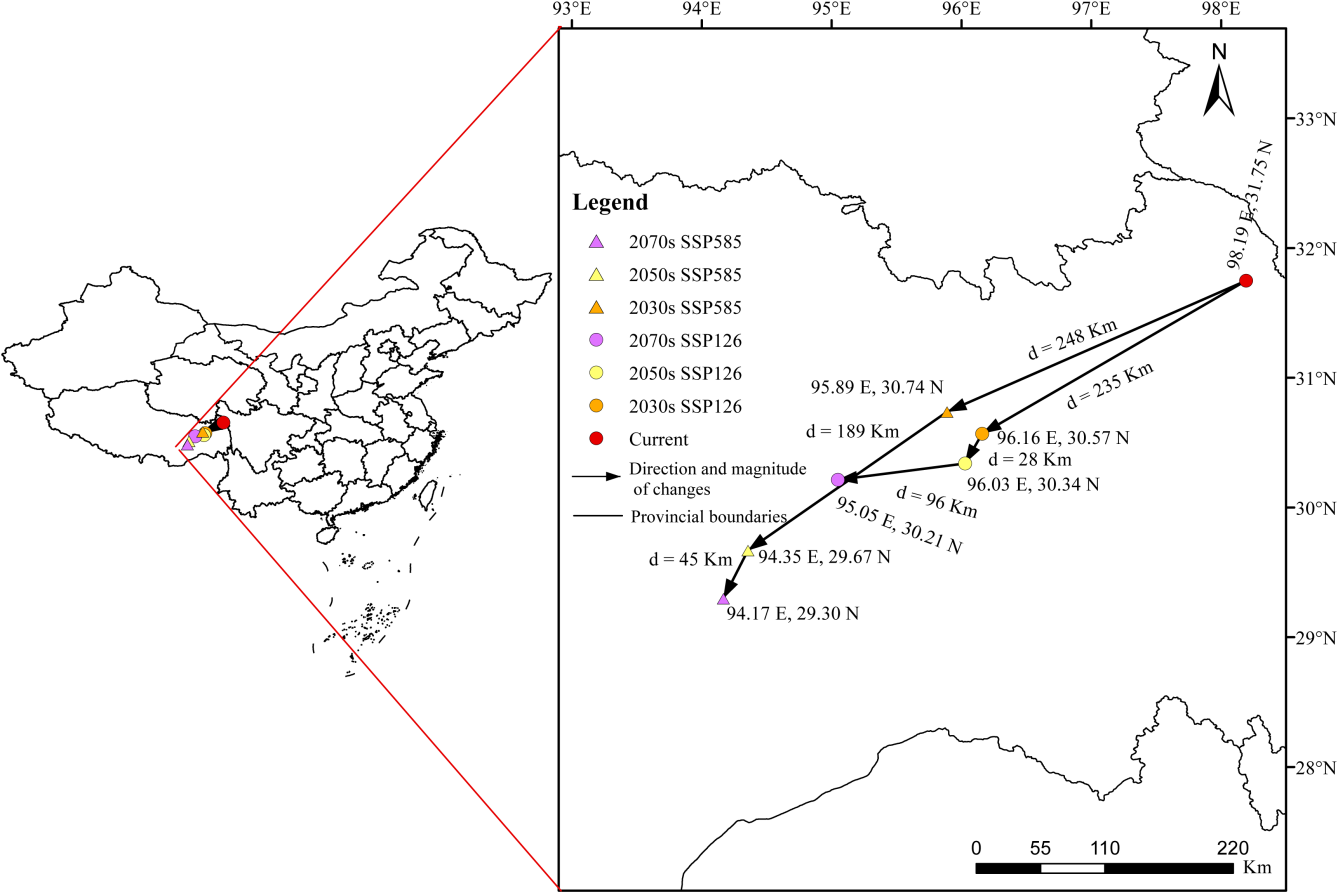
Centroids shifts of *Saussurea medusa* under future climate change scenarios.

To obviously compare the distribution dynamics of the highly suitable habitats of *S. medusa*, the shifts of distribution centroids among different periods and climate scenarios were analyzed and shown in **Figure 8**. In the current projection, the is located in East Xizang near Sichuan, at coordinates 98.191°E, 31.748°N, with an elevation of 4125 meters. In the ssp126 scenario, the distribution centroids were projected to always shift southwestward from 2030s to 2070s. The distances were 235 km, 28 km, and 96 km, representing the three period stages, respectively. At the 2070s, the coordinates of distribution centroid was 95.047°E, 30.214°N, with an elevation of 4116 meters. In the ssp585 scenario, the distribution centroids were projected to always shift southwestward from 2030s to 2070s as well. The distances were 248 km, 189 km, and 45 km, representing the three period stages, respectively. At the 2070s, the coordinates of distribution centroid was 95.166°E, 29.298°N, with an elevation of 4539 meters.

## 4 Discussion

### 4.1 Potential distribution dynamics of *S. medusa* under near current and future conditions

*Saussurea medusa* is a valuable medicinal plant with various pharmacological activities and holds great potential for widespread application in human medicine (Fan et al., 2015). However, this alpine plant faces a survival crisis primarily due to climate change and anthropogenic activities, such as over-harvesting (Zhao et al., 2024). This study, for the first time, predicted the potential distributions of *S. medusa* habitats and identified the underlying driving factors using ensemble models. The potential distribution range of suitable habitats generally covers or even exceeds the sampling points recorded from previous field observations, suggesting that the distribution pattern of *S. medusa* habitat predicted in the present study is reliable.

Under the current climatic conditions, *S. medusa* is primarily distributed across the Qinghai-Xizang Plateau of China, covering the border regions of four provinces: Xizang, Qinghai, Sichuan, and Yunnan. Overall, the predicted suitable regions for *S. medusa* habitats align with those identified by Peng et al. (2019) using the MaxEnt modeling method. However, significant difference was present in the range size. Our findings showed a larger coverage, and even the highly suitable area revealed herein is comparable to the whole suitable area of Peng et al. (2019) in size. Under future climate change scenarios, the entire suitable area is projected to significantly contract, which is consistent with the highly suitable habitats recovered herein. We attribute these differences primarily to variations in model methods and parameters. Firstly, the models used in the studies are different, despite the Maxent method used in Peng et al. (2019) being one of the ensemble models used our study. Secondly, the threshold values defining suitable and unsuitable habitats vary. We adopted the 0.2 threshold value, which is widely used in ensemble models (e.g., Li et al., 2024a; Liu et al., 2022; Zhang et al., 2018), whereas Peng et al. (2019) used the 10 percentile training presence threshold. Thirdly, the different occurrence point datasets for *S. medusa* may also explain the discrepancies. The integrity of input occurrence records within the known distribution range of the modeled species significantly affects the projection results (Sillero et al., 2021; Thibaud et al., 2014). In our analysis, we used 131 occurrence records generally covering the known distribution range of *S. medusa*, compared to the 36 records used in Peng et al. (2019). In summary, our results, derived from ensemble models with more sampling points, may be more effective in revealing the distribution patterns of *S. medusa*.

### 4.2 Impacts of climate and elevation on the distribution of *S. medusa*

Among the ecological factors, climate exerts direct effects on the growth and development of vegetation and is often regarded the dominant factor influencing their geographical distribution of them (Kling et al., 2020; Huang et al., 2024; Li et al., 2024b). In our analyses, eight bioclimatic factors were selected, with three related to temperature and the remaining five to precipitation. Among them, BIO4, BIO18 and BIO12 consistently represent the three top bioclimatic variables contributing to the models, irrespective of whether elevation is included in the variable combinations, indicating that both temperature and precipitation have significant impacts on the distribution of *S. medusa*.

The elevation factor, frequently associated with temperature and water availability, can act as a dispersal barrier and is crucial in shaping the distribution of species (Azrag et al., 2018; Chardon et al., 2015; Zhang et al., 2022). Alpine plants, for instance, often display a “sky island” distribution model, where populations are isolated by steep valleys due to adaptations to extreme environments such as cold temperatures, high UV radiation, and low oxygen levels (Wang et al., 2009; Zhang et al., 2023). Furthermore, they are considered particularly sensitive to the effects of global warming (Korner, 2003). With climate warming, the suitable range for *Saussurea* species shifts to higher elevations, but the restricted space in alpine regions often increases the risk of extinction (Peng et al., 2019; Zhao et al., 2024). In our analysis, elevation was the most significant factor in the modeling, accounting for up to 33.18%, which aligns with Zhao et al. (2024), who modeled other *Saussurea* species using the MaxEnt algorithm. Additionally, the optimal threshold for elevation was 2580–5600 meters, generally consistent with Liu (1996) based on field observations. Moreover, under future climate change scenario, our analysis also revealed shifts in the distribution range to higher elevations as founded in other *Saussurea* species (Zhao et al., 2024). The distribution centroid of the highly suitable habitats in the current projection is located in East Xizang near Sichuan, at coordinates 98.191°E, 31.748°N, with an elevation of 4125 meters. For both two climatic scenarios, the distribution centroids would shift southwestward in Xizang. At ssp126 and ssp585 of 2070s, the coordinates of distribution centroids were 95.047°E, 30.214°N, with an elevation of 4116, and 95.166°E, 29.298°N, with an elevation of 4539 meters, respectively.

### 4.3 Implications for *S. medusa* conservation

The shrinking of suitable habitats, especially the highly suitable habitats, under climate warming indicates effective protection should be consistently conducted. Overall, the National Nature Reserves established before exhibit a relatively high coverage with the suitable habitats of *S. medusa*, such as those in West Sichuan and Southeast Qinghai, suggesting they could provide extensive protection for *S. medusa.* However, there is a significant gap in most of East Xizang, where there are few National Nature Reserves but which represent the highly suitable habitats of *S. medusa* for both current and future periods. This suggests a need for enhanced protection measures, especially in this region. On one hand, designating protected areas in this region, also based on comprehensive investigations on the diversity and conservation needs of other *Saussurea* species or alpine plantss (Zhao et al., 2024). On the other hand, long-term ecological monitoring on the population dynamics of *S. medusa* should be conducted, providing a scientific basis for targeted conservation measures (Zhao et al., 2024). Finally, considering that over-harvesting by humans is a primary cause of the decline in the *S. medusa* population, it is crucial to enhance public education and increase awareness about the protection of *S. medusa* and its sustainable development.

### 4.4 Limitations and future prospects

Using the ensemble models that has never been used before for the important medicinal plant *S. medusa*, we predicted the spatiotemporal distribution patterns and further proposed the preliminary conservation strategies. Our findings are reliable, as evidenced by model evaluation and comparison with results from other studies (e.g., Peng et al., 2019; Zhao et al., 2024). In the present study, although climate-associated variables and elevation are considered, biological factors such as species interactions and human activities were not included. Given that the distribution patterns of a species are shaped by multiple factors, some biotic and abiotic factors are increasingly suggested to be jointly considered with traditionally used climate variables in SDM studies (Li et al., 2024a; Liu and Chen, 2024; Neupane et al., 2024). Future predictions should incorporate additional factors (e.g., human footprint and soil properties) that influence *S. medusa* growth to refine the distribution pattern, potentially providing more accurate guidance for *S. medusa* conservation.

## 5 Conclusions

This study simulated an ensemble model to predict the potential spatiotemporal distribution patterns of an endangered medicinal plant, *S. medusa*, and identified its underlying driving factors. The findings show that the current suitable habitats of *S. medusa* are primarily distributed across the Qinghai-Xizang Plateau in China. Under future climate warming scenarios, the area of suitable habitats, especially the highly suitable ones, is projected to contract significantly by 80.65%, accompanied by shifts in distribution centroids towards southwest and higher altitudes. Among the ecological factors analyzed, elevation and precipitation play a significant role in influencing the habitat distribution of *S. medusa*. The results imply that the risk to the survival of *S. medusa* would persist in the future, and conservation efforts for *S. medusa*, such as the establishment of protected areas and public education, should be undertaken.

## Data Availability

The original contributions presented in the study are included in the article/**Supplementary Material**. Further inquiries can be directed to the corresponding authors.

## Supplementary material

Supplementary Figure 1 The initial occurrence records of *Saussurea medusa* Maxim gathered from four sources.

Supplementary Figure 2 The Pearson’s correlation analysis among 19 bioclimatic variables.

Supplementary Figure 3 The area under the receiver operating characteristic curve (AUC) and true skill statistics (TSS) values for six single models under BIOs + elevation.

Supplementary Table 1 The initial occurrence records of *Saussurea medusa* gathered from four sources.

Supplementary Table 2 The occurrence records of *Saussurea medusa* used in the models.
