# Supplementary figures and images for: Predicting habitat suitability for an endangered medicinal plant, Saussurea medusa: insights from ensemble species distribution models
Source: Front Plant Sci. 2025 Jun 24;16:1590206. doi: 10.3389/fpls.2025.1590206 (PMC12236372; doi:10.3389/fpls.2025.1590206)

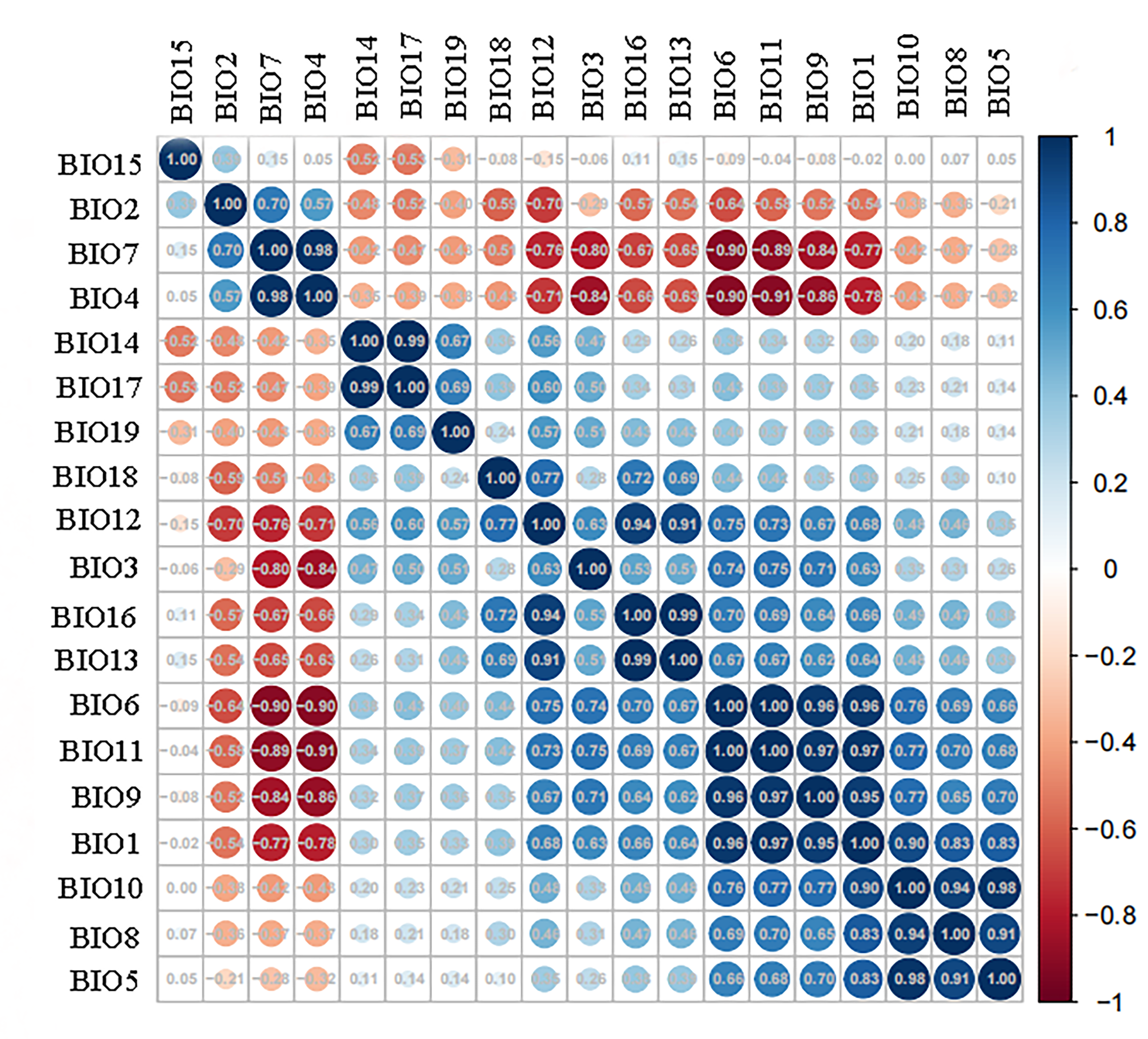

Supplement: Supplementary Figure 1 — The initial occurrence records of Saussurea medusa Maxim gathered from four sources. [file Image1.tif]

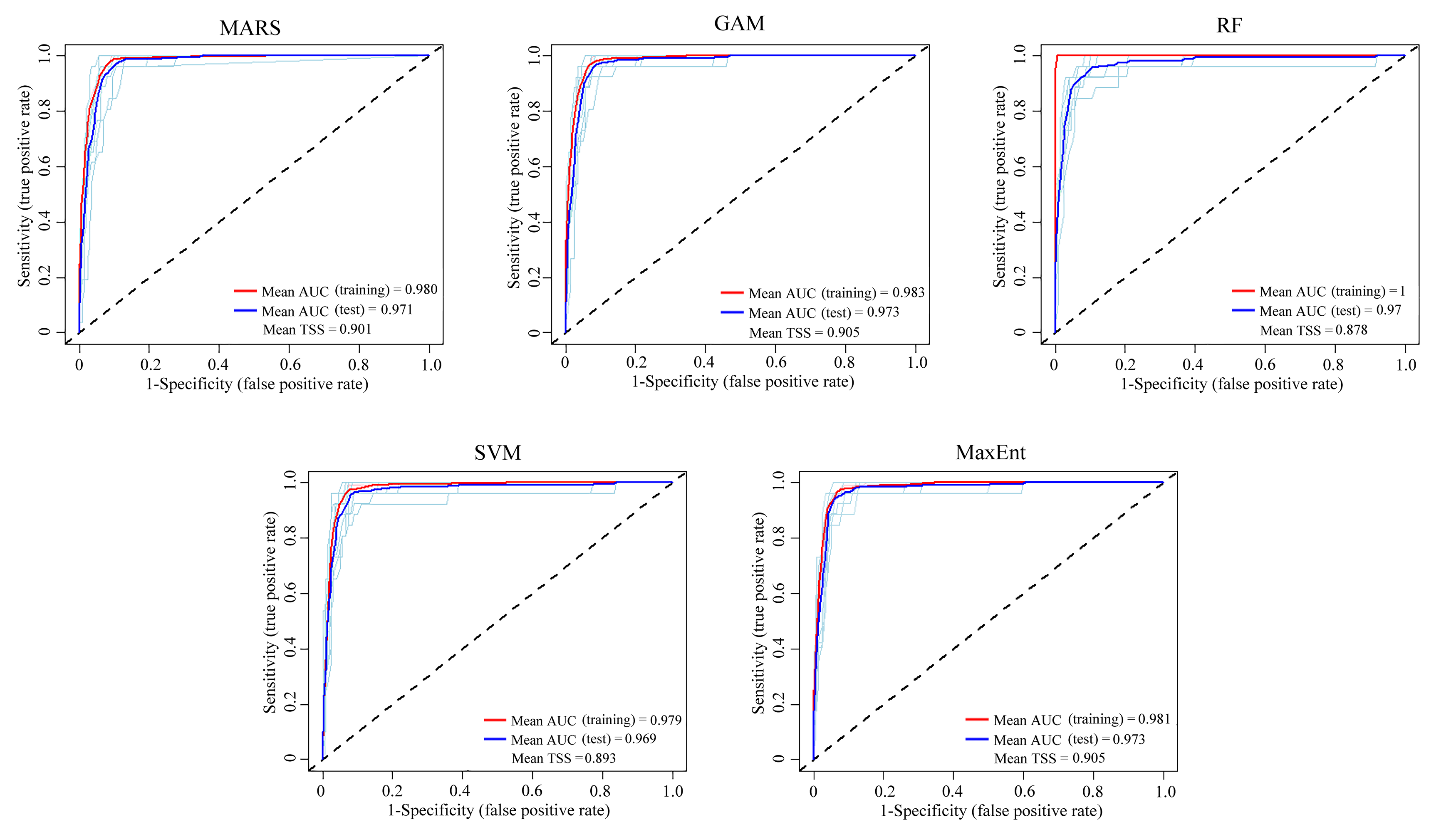

Supplement: Supplementary Figure 2 — The Pearson’s correlation analysis among 19 bioclimatic variables. [file Image2.tif]
